# Accumulation of FOXP3+T-cells in the tumor microenvironment is associated with an epithelial-mesenchymal-transition-type tumor budding phenotype and is an independent prognostic factor in surgically resected pancreatic ductal adenocarcinoma

**DOI:** 10.18632/oncotarget.2775

**Published:** 2015-02-10

**Authors:** Martin Wartenberg, Inti Zlobec, Aurel Perren, Viktor Hendrik Koelzer, Beat Gloor, Alessandro Lugli, Karamitopoulou Eva

**Affiliations:** ^1^ Clinical Pathology Division, University of Bern, Bern, CH-3010, Switzerland; ^2^ Translational Research Unit, Institute of Pathology, University of Bern, Bern, CH-3010, Switzerland; ^3^ Department of Visceral Surgery, Insel University Hospital, Bern, CH-3010, Switzerland

**Keywords:** FOXP3, CD8, Tumor-associated macrophages, pancreatic cancer, prognosis, immune cell infiltration, epithelial mesenchymal transition, tumor budding, tumor microenvironment

## Abstract

Here we explore the role of the interplay between host immune response and epithelial-mesenchymal-transition (EMT)-Type tumor-budding on the outcome of pancreatic adenocarcinoma (PDAC).

CD4+, CD8+, and FOXP3+T-cells as well as iNOS+ (M1) and CD163+-macrophages (M2) were assessed on multipunch tissue-microarrays containing 120 well-characterized PDACs, precursor lesions (PanINs) and corresponding normal tissue. Counts were normalized for the percentage of tumor/spot and associated with the clinico-pathological features, including peritumoral (PTB) and intratumoral (ITB) EMT-Type tumor-budding and outcome.

Increased FOXP3+T-cell-counts and CD163-macrophages and decreased CD8+T-cell-counts were observed in PDACs compared with normal tissues and PanINs (*p* < 0.0001). Increased peritumoral FOXP3+T-cell-counts correlated significantly with venous invasion, distant metastasis, R1-status, high-grade ITB, PTB and independently with reduced survival. Increased intratumoral FOXP3+T-cells correlated with lymphatic invasion, N1-stage, PTB and marginally with adverse outcome. High peritumoral CD163-counts correlated with venous invasion, PTB and ITB. High intratumoral CD163-counts correlated with higher T-stage and PTB.

PDAC-microenvironment displays a tumor-favoring immune-cell composition especially in the immediate environment of the tumor-buds that promotes further growth and indicates a close interaction of the immune response with the EMT-process. Increased peritumoral FOXP3+T-cell density is identified as an independent adverse prognostic factor in PDAC. Patients with phenotypically aggressive PDACs may profit from targeted immunotherapy against FOXP3.

## INTRODUCTION

PDAC is a highly lethal malignancy refractory to standard therapies and characterized by a striking desmoplastic reaction of the stromal compartment [1, 2]. A recent report from the Pancreatic Cancer Action Network estimates that by 2020, PDAC will become the second leading cause of cancer-related death [3, 4]. Despite recent advances with combination chemotherapy to date PDAC remains a significant medical problem that requires an innovative and novel therapeutic approach [5].

The tumor microenvironment plays an important role in the biological behavior of cancer [6] and the host immune reaction, as represented by the peritumoral and intratumoral immune cell infiltrates, is one of its main players [7–12]. Although supposed to be a manifestation of the immune response against neoplastic cells [13, 14], the presence of immune cell infiltrates in the microenvironment of various cancers does not always have a positive influence on patient outcome. Especially FOXP3+T-cells and M2-polarized macrophages have been shown to have an anti-inflammatory function and to suppress anti-tumor immunity thus promoting tumor cell survival [15, 16].

In many gastrointestinal carcinomas, including pancreatic cancer, epithelial-mesenchymal transition (EMT), histomorphologically represented by the presence of tumor budding, is a hallmark of aggressive behavior [17–20]. Moreover, it is thought that tumor buds may possess stem-cell like features and represent migrating cancer cells [21]. Tumor budding was inversely correlated to CD8+ T-cell counts at the advancing edge of colorectal cancer, suggesting that T-cell infiltrates may represent a defense mechanism against tumor budding cells [22]. However, little is known on the interaction between tumor buds and the immune response in the microenvironment of PDAC.

To address this issue, we performed an analysis of the immune cell infiltrates in the microenvironment of surgically resected PDACs in correlation with EMT-Type tumor budding and other clinicopathological features, by using multiple punch tissue microarrays. In addition, we compare the immune cell counts in the microenvironment of PDAC with that of precursor lesions (pancreatic intraepithelial neoplasia: PanIN) and of non-neoplastic pancreatic tissue. We hypothesize that an EMT-Type high-grade tumor budding phenotype is associated with privileged immune conditions, conferring to budding cells a survival benefit by evading the host defense. The combined assessment of the host immune response with factors of tumor aggressiveness like tumor budding could help us to achieve superior prognostic stratification of the patients than either factor alone.

## RESULTS

### Patient characteristics

Tissues from 120 patients with PDAC were included in the tissue microarray. Study design is outlined in Suppl. Figure 1. Median overall survival (OS) for the cohort of 120 patients was 12.9 months (95%CI: 10–13), while the median disease-free interval (DFI) was 5.9 months (95%CI: 4–6). Patient characteristics are outlined in Suppl. Table 1.

### Normal-PanIN-Carcinoma sequence

**Figure 1:**
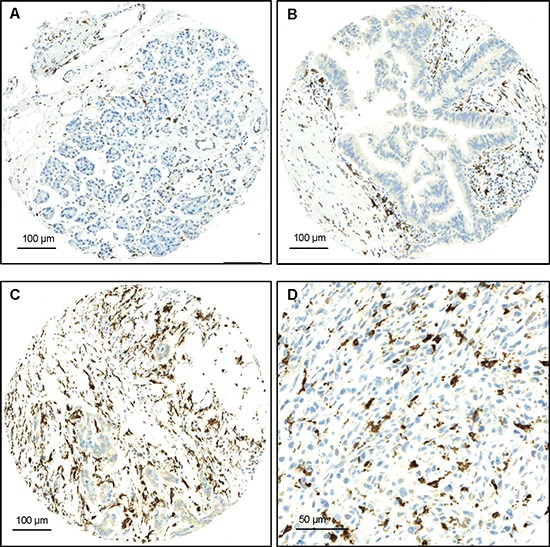
Examples of FOXP3 staining **(A)** Normal pancreatic tissue with low counts of FOXP3+ T-cells, x100 (Bar:100μm); **(B)** PanIN with moderate FOXP3+-T-cell infiltrates, x100 (Bar:100μm); **(C)** PDAC with numerous peritumoral FOXP3+T-cells x100 (Bar:100μm); **(D)** PDAC with numerous peritumoral FOXP3+T-cells, x400 (Bar:50μm).

**Figure 2:**
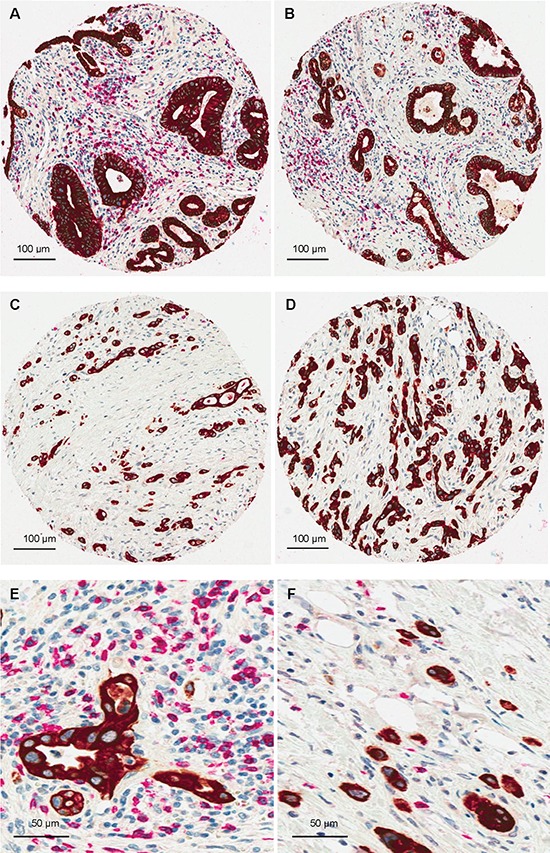
CD8/Pancytokeratin double staining demonstrating a strong negative correlation between EMT-type tumor budding and CD8+T-cells **(A and B)** Examples of PDAC with numerous peritumoral CD8+T-cells and absence of tumor buds, x100 (Bar:100 μm); **(C and D)** PDACs with high-grade tumor budding and markedly reduced peritumoral CD8+T-cell counts, x100 (Bar:100 μm); **(E)** PDAC with numerous peritumoral CD8+T-cells and absence of tumor buds, x400 (Bar:50μm); **(F)** PDAC with high-grade tumor budding and markedly reduced peritumoral CD8+T-cell counts, x400 (Bar:50 μm).

A significant progressive increase in overall FOXP3+T-cell-counts and CD163-macrophages (M2) was found between normal pancreatic tissue, PanINs and PDACs (*p* = 0.0114, Figure 1, Table 1). The opposite was true for the CD8+T-cell infiltrates which were found to be markedly decreased in PDACs compared with normal tissues and PanINs (*p* < 0.0001, Table 1). These differences were more noticeable when taking into account the peritumoral/perilesional cell counts (p). Intraepithelial immune cell counts (i) were in general very low (Table 1). In PDACs a strong negative correlation between peritumoral CD8+T-cell counts and tumor budding was observed (*p* = 0.01, Figure 2).

**Table 1:** Differences in average cell counts of the different markers across histology

	Normal	PanIN	PDAC	*P*-value*
	Average No.	Average No.	Average No.	
**CD8p**	107.6	90.3	58.1	0.0114
**CD8i**	13	4.2	0.6	<0.0001
**CD4p**	14.2	57.8	22.5	<0.0001
**CD4i**	0.09	2.03	0.3	<0.0001
**FOXP3p**	3.6	7.1	10.9	<0.0001
**FOXP3i**	0	0.12	0.5	<0.0001
**CD163 (M2)p**	115.1	140	153.4	0.0016
**CD163 (M2)i**	1.1	3.16	3.6	<0.0001
**iNOS (M1)p**	4.32	1	0.38	<0.0001
**iNOS (M1)i**	0.17	0	0	0.006

PanIN: Pancreatic Intraepithelial Neoplasia; PDAC: Pancreatic Ductal Adenocarcinoma; p: pericellular/perilesional/and/or peritumoral localisation; i: intracellular/ntralesional/ and/or intratumoral localisation

### Association with clinicopathological features

**Figure 3:**
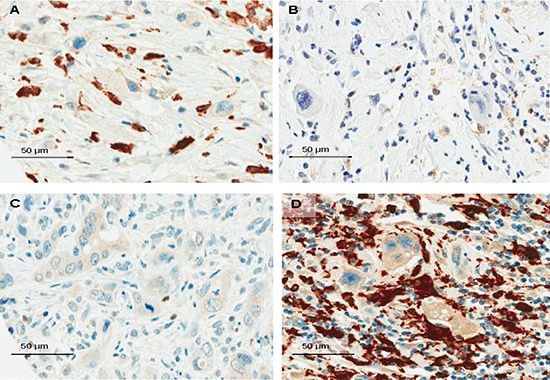
Snapshots of the tumor microenvironment of PDAC demonstrating tumor budding cells surrounded by numerous FOXP3+T-cells (A, x400, Bar:50μm); moderate counts of CD4+T-cells (B, x400, Bar:50μm); isolated iNOS-macrophages (C, x400, Bar:50μm) and dense infiltrates of CD163-macrophages (D, x400, Bar:50μm)

Results for all markers for associations with clinicopathological features when considering all patients and using the median normalized cell counts are summarized in Suppl. Table 2. Representative examples of the immune cell infiltrates in the microenvironment of PDAC are depicted in the Figures 2 and 3. Increased peritumoral FOXP3+T-cell-counts (FOXP3p) correlated with high-grade tumor budding including budding 10-in-10 (*p* = 0.0425) previously assessed in whole tissue sections, as well as ITB (*p* = 0.0428) and PTB (*p* < 0.0001), assessed at the TMA spots for each protein and showed significant correlation with the presence of venous invasion (*p* = 0.0189), distant metastasis (*p* = 0.0073) and positive resection margins (*p* = 0.0067) (Table 2). Increased intratumoral FOXP3+T-cells correlated with lymphatic invasion (*p* = 0.0062), N-stage (*p* = 0.0022) and PTB (*p* = 0.0041, Table 2). High peritumoral CD163-counts (CD163p) correlated with venous invasion (*p* = 0.0291), budding 10-in-10 (*p* = 0.0155), PTB (*p* = 0.0200) and ITB (*p* = 0.0253, Table 3). High intratumoral CD163-counts (CD163i) correlated significantly with T-stage (*p* = 0.006) and PTB (*p* = 0.0349, Table 3).

**Table 2:** Association of FOXP3 positive cells, peritumoral (p) and intratumoral (i) and clinicopathological features in PDAC

Feature		FOXP3p			FOXP3i		
		Freq N (%)		*P*-value	Freq N (%)		*P*-value
		Low	High		Low	High	
**Gender**	Female	21 (36.2)	27 (54.0)	0.0635	24 (43.6)	24 (45.3)	0.8633
	Male	37 (63.8)	23 (46.0)		31 (56.4)	29 (54.7)	
**Grade**	G1-2	8 (13.6)	7 (14.0)	0.9469	7 (12.5)	8 (15.1)	0.6943
	G3	51 (86.4)	43 (86.0)		49 (87.5)	45 (84.9)	
**pT**	pT1-2	5 (8.6)	6 (12.2)	0.5385	6 (11.1)	5 (9.4)	0.7752
	pT3	53 (91.4)	43 (87.8)		48 (88.9)	48 (90.6)	
**pN**	pN0	8 (13.8)	10 (20.4)	0.4402	15 (27.8)	3 (5.7)	**0.0022**
	pN1	50 (86.2)	39 (79.6)		39 (72.2)	50 (94.3)	
**pM**	pM0	50 (86.2)	49 (100.0)	**0.0073**	51 (94.4)	48 (90.6)	0.4886
	pM1	8 (13.8)	0 (0.0)		3 (5.6)	5 (9.4)	
**L**	L0	11 (19.0)	8 (16.3)	0.7219	15 (27.8)	4 (7.6)	**0.0062**
	L1	47 (81.0)	41 (83.7)		39 (72.2)	49 (92.5)	
**V**	V0	57 (95.0)	38 (77.6)	**0.0189**	46 (85.2)	39 (73.6)	0.1377
	V1	3 (5.0)	11 (22.5)		8 (14.8)	14 (26.4)	
**R**	R0	34 (59.7)	41 (83.7)	**0.0067**	35 (66.0)	40 (75.5)	0.1954
	R1	23 (40.3)	8 (16.3)		18 (34.0)	13 (24.5)	
**Chemotherapy**	None	1 (1.9)	2 (4.2)	0.6031	1 (1.9)	2 (4.2)	0.6031
	Yes	52 (98.1)	46 (95.8)		52 (98.1)	46 (95.8)	
**Radiotherapy**	None	20 (74.1)	40 (87.0)	0.2098	22 (78.6)	38 (84.4)	0.5441
	Yes	7 (25.9)	6 (13.0)		6 (21.4)	7 (15.6)	
**Budding 10-in-10**	Low	20 (37.0)	10 (20.8)	**0.0425**	17 (34.7)	13 (24.5)	0.2843
	High	34 (63.0)	38 (79.2)		32 (65.3)	40 (75.5)	
**ITB (per punch)**	Low	30 (52.6)	16 (32.0)	**0.0428**	26 (47.3)	17 (32.7)	0.1674
	High	27 (47.4)	34 (68.0)		29 (52.7)	35 (67.3)	
**PTB (per punch)**	Low	36 (65.5)	13 (25.0)	**<0.0001**	33 (60.0)	16 (30.8)	**0.0035**
	High	19 (34.6)	39 (75.0)		22 (40.0)	36 (69.2)	
**OS**	Median (95%CI)	13 (9–15)	10.5 (9–12)	**0.0027**	12 (10–14)	10 (9–12)	0.0507
**DFI**	Median (95%CI)	5.5 (4–7)	5 (4–6)	0.2522	6 (5–6)	5 (4–6)	0.675

L: lymphatic invasion; V: venous invasion; R: resection margin; ITB: intratumoral budding; PTB: peritumoral budding; OS: overall survival; DFI: Disease Free Interval

**Table 3:** Association of CD163 positive cells, peritumoral (p) and intratumoral (i) and clinicopathological features in PDAC

Feature		CD163p			CD163i		
		Freq N (%)		*P*-value	Freq N (%)		*P*-value
		Low	High		Low	High	
**Gender**	Female	28 (47.5)	23 (46.0)	1.0	24 (44.4)	25 (48.1)	0.7121
	Male	31 (52.5)	27 (54.0)		30 (55.6)	27 (51.9)	
**Grade**	G1-2	6 (10.0)	6 (12.2)	0.7179	4 (7.3)	8 (15.1)	0.4874
	G3	54 (90.0)	33 (87.8)		51 (92.7)	45 (84.9)	
**pT**	pT1-2	8 (13.3)	7 (14.0)	1.0	1 (1.8)	12 (23.1)	**0.0069**
	pT3	52 (86.7)	43 (86.0)		54 (98.2)	40 (76.9)	
**pN**	pN0	13 (21.7)	5 (10.0)	0.2041	10 (18.2)	9 (17.0)	0.8366
	pN1	47 (78.3)	25 (90.0)		45 (81.8)	44 (83.3)	
**pM**	pM0	36 (61.1)	29 (58.0)	1.0	30 (54.5)	29 (54.7)	0.7179
	pM1	23 (38.9)	21 (42.0)		25 (45.5)	24 (45.3)	
**L**	L0	12 (19.7)	3 (6.1)	0.1942	7 (12.7)	9 (16.9)	0.6607
	L1	49 (80.3)	46 (93.9)		48 (87.3)	44 (83.1)	
**V**	V0	47 (78.3)	48 (96.0)	**0.0291**	49 (89.1)	43 (81.1)	0.3567
	V1	13 (21.7)	2 (4.0)		6 (10.9)	10 (19.9)	
**R**	R0	43 (71.6)	37 (75.5)	0.7955	39 (70.9)	41 (77.3)	0.5523
	R1	17 (28.4)	12 (24.5)		16 (29.1)	12 (22.7)	
**Chemotherapy**	None	1 (1.7)	0 (0.0)	1.0	0 (0.0)	1 (1.9)	1.0
	Yes	59 (98.3)	50 (100.0)		55 (100.0)	52 (98.1)	
**Radiotherapy**	None	32 (86.5)	15 (83.3)	1.0	23 (92.0)	24 (80.0)	0.2689
	Yes	5 (13.5)	3 (16.7)		2 (8.0)	6 (20.0)	
**Budding 10-in-10**	Low	13 (21.6)	24 (48.0)	**0.0155**	17 (30.9)	17 (32.1)	1.0
	High	47 (78.4)	26 (52.0)		38 (69.1)	36 (67.9)	
**ITB (per punch)**	Low	19 (31.6)	27 (57.1)	**0.0253**	19 (34.5)	25 (47.1)	0.365
	High	41 (68.4)	22 (42.9)		36 (65.5)	28 (52.9)	
**PTB (per punch)**	Low	24 (40.0)	27 (56.0)	**0.0200**	32 (58.1)	18 (33.9)	**0.0349**
	High	36 (60.0)	23 (44.0)		23 (41.9)	35 (66.1)	
**OS**	Median (95%CI)	10 (9–12)	13 (10–15)	0.0823	12 (9–15)	11 (9–13)	0.562
**DFI**	Median (95%CI)	5 (4–6)	6 (4–8)	0.3054	5 (4–6)	6 (4–6)	0.7789

L: lymphatic invasion; V: venous invasion; R: resection margin; ITB: intratumoral budding; PTB: peritumoral budding; OS: overall survival; DFI: Disease Free Interval

### Prognostic significance

Regarding prognosis, reduced peritumoral CD8+T-cell counts were associated with worse survival time of the patients in the univariate (*p* = 0.0372) but not in the multivariate analysis when adjusted for T-, N-, M-stage, tumor budding and therapy (*p* = 0.8862, Suppl. Table 3).

**Figure 4:**
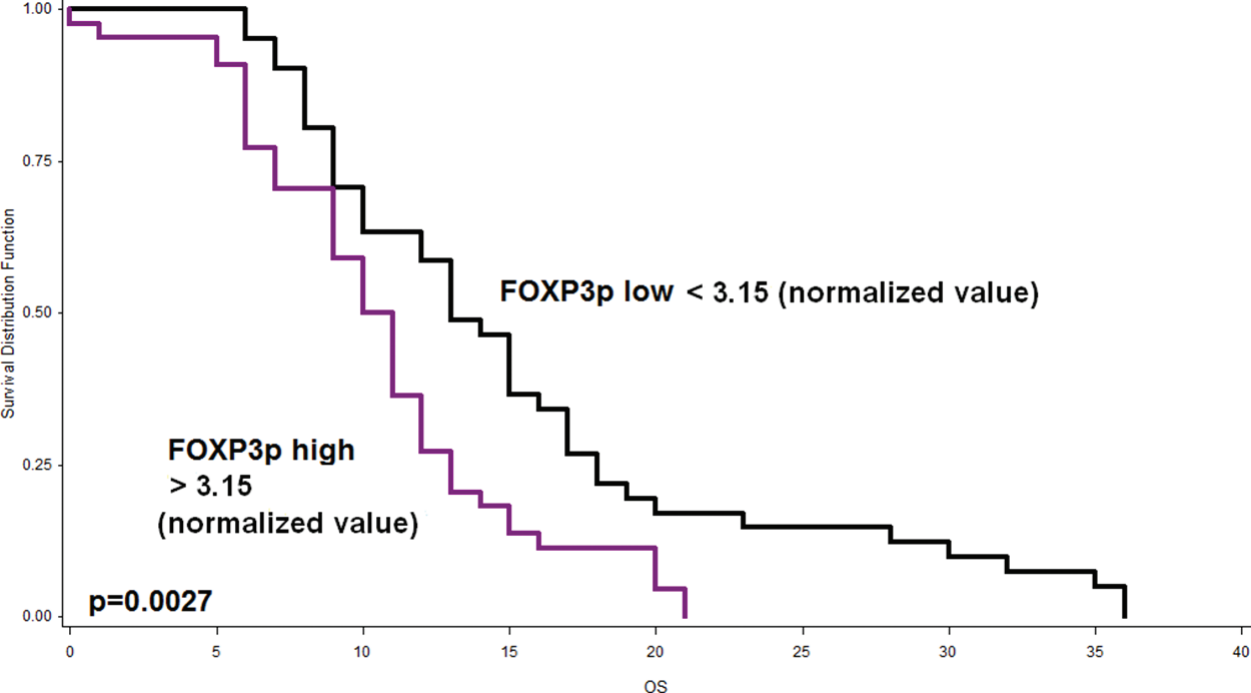
Kaplan-Meier curve indicating the prognostic effect of peritumoral FOXP3+T-cell infiltrates on outcome of PDAC patients Patients with low FOXP3+T-cell counts exhibit significantly longer survival (*p* = 0.027).

On the contrary, increased peritumoral FOXP3+T-cell counts showed a significant association with worse outcome and were found to be an independent prognostic factor in the multivariate analysis, when adjusted for T-, N-, M-stage, tumor budding and therapy (*p* = 0.0027, Table 4, Figure 4) and when adjusted for the other immune cell counts (Suppl. Table 4). Intratumoral FOXP3+T-cell counts showed a marginal association with reduced survival (*p* = 0.0572, Table 2). No association with patient outcome was found for CD4, CD163 and iNOS counts.

**Table 4:** Multivariate analysis of FOXP3p in PDAC patients

		HR (95%CI)	*P*-value
**FOXP3p**	**Low**	1.0	**0.0028**
	**High**	2.03 (1.3–3.2)	
**pT**	**pT1-2**	1.0	0.9557
	**pT3**	1.02 (0.47–2.24)	
**pN**	**pN0**	1.0	0.9418
	**pN1**	1.02 (0.57–1.82)	
**pM**	**pM0**	1.0	**0.0001**
	**pM1**	8.54 (2.9–5.6)	
**Budding**	**Low**	1.0	**0.0002**
	**High**	3.34 (1.78–6.3)	
**Chemotherapy**	**No**	1.0	
	**Yes**	0.05 (0.01–0.3)	**0.0013**

## DISCUSSION

Here we show that the tumor microenvironment of phenotypically aggressive PDAC is characterized by the presence of EMT-Type tumor budding cells surrounded by numerous pro-tumoral leukocytes such as FOXP3+T-cells and CD163+ (M2-polarized) macrophages with concomitant reduction of anti-tumoral immune cell populations such as CD8+T-cells and iNOS+ (M1-polarized) macrophages, thus creating a privileged immune environment for the further tumor growth and metastasis.

According to Hanahan & Weinberg [6] neoplastic cells are often capable of driving inflammatory pathways that recruit pro-tumoral leukocytes to the tumor microenvironment and can actively evade attack and elimination by the immune host response. This escape of the host immune defense by the neoplastic cells is considered to be an essential step towards metastatic spread. Our data suggests that budding cells may indeed interact with their immunological microenvironment during EMT-process. Thus, tumor budding was found to be particularly prominent when reduced numbers of peritumoral CD8+T-cells were present. On the contrary, when the host was able to maintain a strong anti-tumoral immune response including numerous CD8+T-cells, tumor budding was almost absent. One hypothesis may be that host response is capable of destroying budding cells at the invasive front. Alternatively, this could signal the existence of a budding cell population with more antigenic phenotype that has yet to acquire the ability to evade immunosurveillance. Furthermore, our results are partly in line with previous studies that showed that tumor infiltration by higher numbers both of CD4+T-cells and CD8+T-cells was associated with longer survival of PDAC patients [23]. However, in the present study, increased counts of peritumoral CD8+T-cells were associated with better prognosis in univariate but not in the multivariate analysis, indicating that increased CD8+ infiltrates alone are not sufficient for a better outcome. CD4+T-cell counts did not have an impact on survival in our study.

A high density of tumor-infiltrating FOXP3 Tregs has been associated with poor outcome in various solid tumors, including ovarian [24, 25], pancreatic [26] and hepatocellular carcinoma [27, 28]. In the present study, high-grade tumor budding was associated with increased counts of peritumoral FOXP3+T-cells. Moreover, FOXP3+T-cells added independent prognostic information for PDAC patients in a multivariate model including other established and independent prognostic factors such as tumor budding. This could be explained by the proposed role of these cells in suppressing anti-tumor immunity and in helping tumor cells escape detection by the host's immune defense system [29]. Moreover, in line with previous reports [26], the prevalence of FOXP3+T-cells increased significantly during the neoplastic progression. Taken together and in consistence with previous data, we identify a significant survival advantage and favourable histopathological features, such as absence of an EMT-type tumor budding, in PDAC patients with CD8+/FOXP3- phenotype, suggesting a close interaction of the immune cells with the molecular pathways that control the EMT process.

Although the role of the innate immune cells of the myeloid lineage such as tumor-associated macrophages (TAMs) in the tumor development has been recognized, only few previous reports indicate a role of macrophages in EMT [30–32]. Our results, by demonstrating a strong association between CD163 + (M2) macrophages and tumor budding, support an EMT-promoting role for M2-macrophages. Activated macrophages can be divided into M1 and M2 phenotype [33] even if it is clear that these are two extremes of a spectrum of differentiation. While M1 macrophages mediate resistance against tumors and elicit tissue disruptive reactions, M2 macrophages have an immune suppressive role [34, 35]. Especially in pancreatic cancer high numbers of M2 macrophages were associated with larger tumor size, local recurrence and shortened survival [36]. Kurahara and colleagues [33] described an elevated incidence of M2 macrophages in PDAC tissues, correlating with increased nodal lymphangiogenesis and poor prognosis partially due to accelerated lymphatic metastasis, but the mechanism by which these cells influence the progression of pancreatic cancer is not yet clear. Our findings suggest that a possible mechanism could be through the promotion of EMT-type tumor budding. Moreover, recent evidence suggests that the phenotype of TAMs varies with the stage of tumor progression. During cancer progression, macrophages switch from an M1- to an M2-like phenotype as the tumor begins to invade, vascularize and develop [37–39]. In agreement with this, we find a progressive decrease of iNOS+ (M1) and an increase of CD163+ (M2) macrophages from the normal tissue, to PanINs and to invasive cancer. Taken together our findings underline the role of TAMs in the pancreatic tumorigenesis and provide support for their association with the process of EMT.

Our results should be understood in the context of the study limitations. Although TMAs provide an efficient and cost-effective tool for testing a comprehensive panel of potential biomarkers on a large number of tumor specimens, the TMA technique could raise concerns related to the sampling of large, heterogeneous tumors. The effect of tumor heterogeneity was minimized by sampling at least two punches from the center and two from the invasive front and evaluating the average protein expression across the total number of samples. Our study may further be limited by the fact that all cases come from a single center. Nonetheless, our study benefits from complete clinicopathological data with information on adjuvant therapy and follow-up and the adherence to the REMARK guidelines which are essential for proposing prognostic biomarkers [40].

In conclusion, our findings suggest that in the tumor microenvironment of PDAC, EMT-type budding cells are surrounded by a tumor-favoring composition of the immune cell infiltrates, promoting further tumor growth and metastatic spread and indicating a close interaction of the immune response with the EMT process. Increased peritumoral FOXP3+T-cell density could be identified as an independent adverse prognostic factor in PDAC. Patients with resected, phenotypically aggressive PDACs may thus profit from a postoperative targeted immunotherapy against FOXP3. The combined assessment of host-associated factors such as immune response and tumor-associated factors such as EMT-type tumor budding could help us to achieve superior prognostication and patient stratification than either factor alone.

## MATERIALS AND METHODS

### Patients and specimen characteristics

This study complies with the REMARK guidelines for tumor marker prognostic studies [40]. The study design is outlined in Figure 1. Non-consecutive PDAC cases from 120 patients, surgically treated between 2000 and 2010, were included. Paraffin-embedded tissue blocks of primary tumors were retrieved from the Department of Pathology, Aretaieion University Hospital, University of Athens Medical School, Greece. All histomorphological data were reviewed from the corresponding hematoxylin and eosin (H&E) stained slides, while clinical data were obtained from chart reports. Clinicopathological information for all patients included age, gender, tumor diameter, number of positive lymph nodes and total number of lymph nodes harvested, TNM stage (7th Edition), perineural, as well as blood vessel and lymphatic invasion and resection margin status (R-status). Information on post-operative therapy was available for all patients (Suppl. Table 1). The use of this material was approved by the local ethics committees of the University of Athens and University of Bern (Ref.-Nr. KEK-BE: 200/2014).

### Assay methods

### Construction of tissue microarrays (TMA)

For each patient, the hematoxylin and eosin slides of the primary tumor from the corresponding whole tissue sections were evaluated and representative areas of the tissue were marked using a felt-tip pen for easy detection. Punches were taken from formalin-fixed, paraffin-embedded blocks using a tissue cylinder with a diameter of 0.6 mm and were subsequently transferred into 1 recipient paraffin block (3 × 2.5 cm) using a homemade semiautomated tissue arrayer. To exclude bias because of possible tumor heterogeneity, each patient had 4 tumor punches obtained from the tumor center and the invasive tumor front (2 tumor center + 2 tumor front) included on this array (total of 480 punches). Two additional one-punch TMAs were constructed including normal pancreatic tissue (147 punches) and precursor lesions (PanINs; 123 punches).

### Immunohistochemistry

TMA blocks were cut at 4 μm and immunostained for CD4 (CD4/4B12, 1:100, pre-treatment with Tris buffer at 95° for 20 minutes; AEC chromogen), and FOXP3 (Abcam, clone 236A/E7, 1:100, pre-treatment in citrate buffer, 30', 100°C) as well as iNOS (iNOS, 1:100, pre-treatment in Tris buffer, 20', 95°C) and CD163 (CD163+; Novocastra, NCL-CD163CD163+;1:100, pre-treatment in Tris buffer, 20', 95°C). Staining was performed using a Bond Max Autostainer (LEICA Bond III platform) from Leica Microsystem (Wetzlar, Germany). Haematoxylin counterstaining was performed. A double immunostaining procedure using anti-CD8 (Dako CD8/144B, 1:100, pre-treatment with Tris buffer at 95° for 20 minutes; AEC chromogen) and pan-cytokeratin (AE1/AE3, Dako, 1:400, pre-treatment with Tris buffer at 100° for 20 minutes) to facilitate visualization of tumor buds at the invasive front was carried out on one representative TMA slide according to a previously described protocol [22].

Negative controls were obtained by staining the slides with an isotype IgG for the same species (ms IgG1 for FOXP3 and CD163, ms IgG1 kappa for CD4 and CD8 and rb IgG for iNOS). No false-positive staining was noted.

### Assessment of tumor budding

Tumor budding was defined as detached single cells or clusters of < 5 cells. Cases have already been evaluated for tumor budding using a 10-in-10 approach by using whole tissue sections immunostained for AE1/AE3 (pan-cytokeratin). Briefly, the 10 densest hot-spots of tumor budding were evaluated at high-magnification (40x, 0.55mm2) and counted. The average number of buds per case was obtained. Using a receiver operating characteristic (ROC) curve approach, a cut-off score of 10 buds on average was identified as most discriminatory for survival. Cases with an average of >10 buds were classified as “high-grade” budders; those with ≤ 10 buds were assigned as “low-grade” budders [20]. Additionally, tumor budding was re-evaluated in the TMAs by counting the number of tumor buds in the punches obtained from the main tumor body (intratumoral budding or ITB) and from the tumor front (peritumoral budding or PTB). The average number of tumor buds was calculated across all punches from the same localization.

### Assessment of immunostaining

The immune cell infiltrates were evaluated by counting the number of positive cells per tissue microarray punch. In the case of multiple tumor punches per localization, the average number was calculated across all punches from the same localization. The end result was that each patient had a final score for the main tumor body, the tumor buds, the precursor lesions (PanINs) and the non-neoplastic pancreatic tissue. Evaluation was performed blinded to clinical endpoints. Counts were normalized for the percentage of tumor/spot and associated with the clinico-pathological features, including peritumoral (PTB) and intratumoral (ITB) (EMT)-Type tumor-budding, outcome and therapy.

### Statistical analysis

In order to determine a valid cut-off score for immune cell counts (low/high), the median normalized values were used (Suppl. Table 5). Association of immune cell counts with categorical clinicopathological features was performed using the Chi-Square test and the Fisher's Exact tests; for continuous variables such as age and tumor size, the non-parametric Wilcoxon's Rank Sum test was used. For matched analyses, the Wilcoxon's Signed Rank test for pairs and the Friedman test for three of more groups were used. Logistic regression analysis was used to determine the odds ratio (OR) and 95%CI with clinicopathological features. Missing data were few and were assumed to be missing at random. No imputation for missing values was performed. Univariate survival time analysis was performed using the log-rank test and differences plotted using Kaplan-Meier curves. *P*-values < 0.05 were considered statistically significant. Correction for multiple hypothesis testing was not carried out [41]. Analyses were carried out using SAS (V9.2; The SAS Institute, Cary, NC).

## SUPPLEMENTARY FIGURE AND TABLES


