# Supplementary material for: Accumulation of FOXP3+T-cells in the tumor microenvironment is associated with an epithelial-mesenchymal-transition-type tumor budding phenotype and is an independent prognostic factor in surgically resected pancreatic ductal adenocarcinoma
Source: Oncotarget. 2015 Feb 10;6(6):4190–201. doi: 10.18632/oncotarget.2775 (PMC4414182; doi:10.18632/oncotarget.2775)
Supplement: Supplementary file 1 [file oncotarget-06-4190-s001.pdf]

SUPPLEMENTARY FIGURE AND TABLES

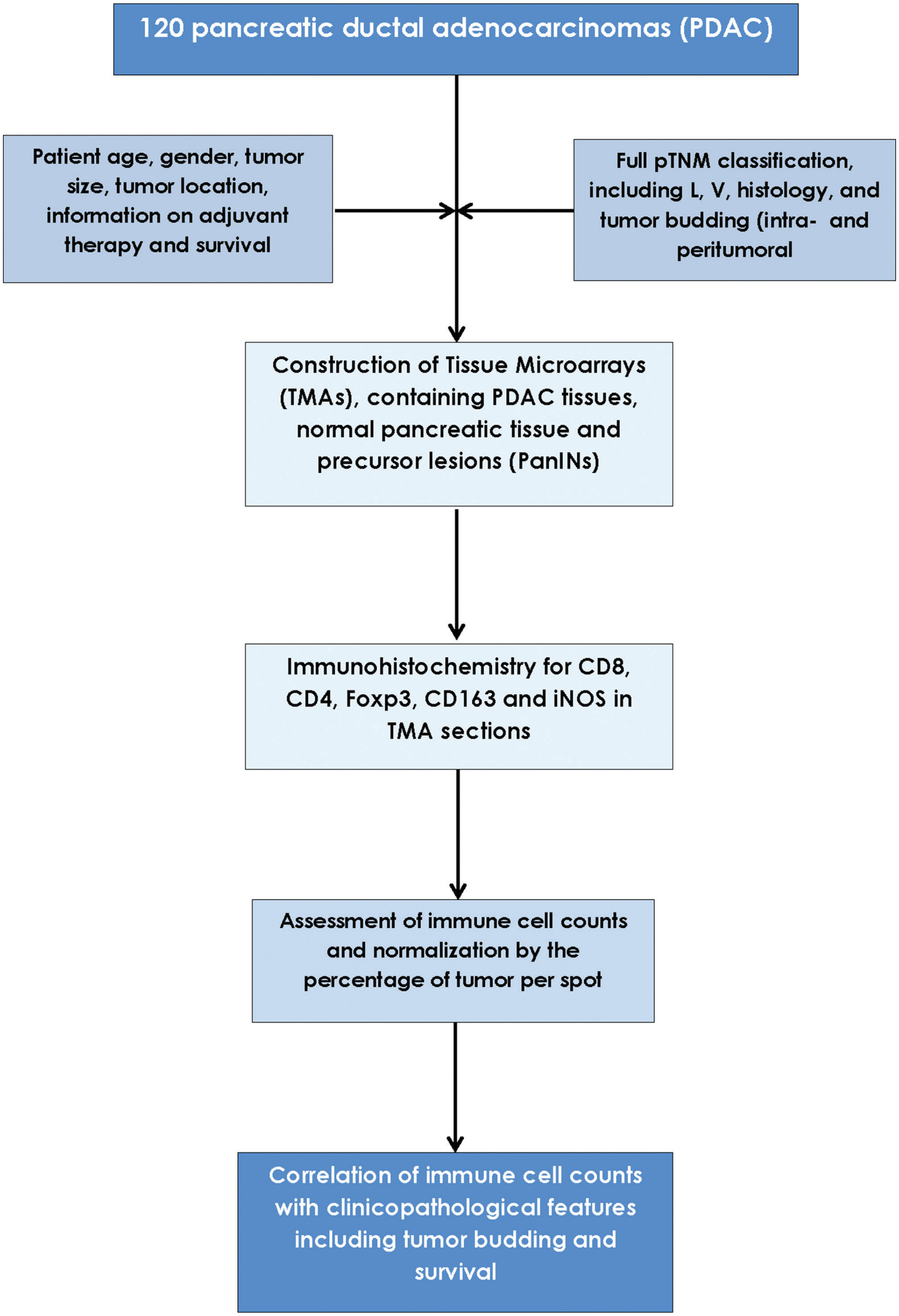

Supplementary Figure 1: Study Design. 120 PDAC patients with full clinicopathological information were entered into the study. Using a multi-punch tissue microarray, the association of CD8+ /CD4+ / and FOXP3+ T-cell infiltration, as well as CD163 (M1) and iNOS (M2) macrophages with EMT-Type tumor budding, prognostic factors and survival was analysed.

Supplementary Table 1: Patient characteristics

Feature	Frequency N (%)
**Age (yrs)**	64.0 (34–84)
**Gender (*n* = 117)**	
**Female**	53 (45.3)
**Male**	64 (54.7)
**Grade (*n* = 115)**	
**G1**	16 (13.7)
**G2**	55 (47.0)
**G3**	46 (39.3)
**Tumor Size (cm)**	3.5 (1.2–10.0)
**No. Positive Lymph Nodes**	
**Mean (min, max)**	2.9 (0–11)
**Total Harvested No. Lymph Nodes**	
**Mean (min, max)**	13.2 (6–37)
**pT (*n* = 115)**	
**T1-2**	11 (9.6)
**T3-4**	104 (91.4)
**pN(*n* = 115)**	
**N0**	21 (18.3)
**N1**	94 (81.7)
**pM(*n* = 115)**	
**M0**	105 (91.3)
**M1**	10 (8.7)
**TNM Stage (*n* = 115)**	
**I**	3 (2.6)
**IIA**	17 (14.8)
**IIB**	81 (70.4)
**III**	4 (3.5)
**IV**	10 (8.7)
**Pn- classification (*n* = 113)**	
**Pn 0**	1 (0.9)
**Pn 1**	112 (99.1)
**L- classification (*n* = 115)**	
**L0**	22 (19.1)
**L1**	93 (80.9)

(*Continued*)

Feature	Frequency N (%)
**V- classification (*n* = 115)**	
**V0**	90 (78.3)
**V1**	25 (21.7)
**R-status (*n* = 114)**	
**R0**	58 (50.9)
**R1**	56 (49.1)
**Neoadjuvant Chemotherapy (*n* = 108)**	
**No**	3 (2.8)
**Yes**	105 (97.2)
**Radiotherapy (*n* = 77)**	
**No**	63 (81.8)
**Yes**	14 (18.2)
**Disease-free interval (months) (*n* = 69)**	
**Months (range)**	5.9 (0–19)
**Overall survival (months) (*n* = 98)**	
**Months (range)**	12.6 (0–36)

Pn-classification: perineural invasion; L-classification: lymphatic invasion; V-classification: venous invasion; R-status: resection margin status

Supplementary Table 2: Summary of *p*-values for associations using Chi-Square or Fisher's Exact test, using the median normalized cell counts

	CD8p	CD8i	CD4p	CD4i	Foxp3p	Foxp3i	CD163p	CD163i	iNOSp	iNOSi
**Gender**	0.7002	0.3627	0.2902	0.8027	0.0635	0.8633	1.0000	0.7121	0.5330	0.1970
**pT-stage**	0.7349	1.0000	0.20	0.7409	0.5385	0.7752	1.0000	**0.0069**	0.1071	0.2237
**Grade**	0.0935	1.0000	0.7567	0.2052	0.9469	0.6943	0.7179	0.4874	0.7729	0.2744
**pN-Stage**	0.4469	0.7317	1.0000	1.0000	0.4402	**0.0022**	0.2041	0.8366	1.0000	0.3686
**V-Stage**	0.1439	0.1057	0.4127	0.6088	**0.0189**	0.1377	**0.0291**	0.3567	0.3822	1.0000
**L-Stage**	0.3737	1.0000	0.5871	0.6779	0.7219	**0.0062**	0.1942	0.6607	0.2013	0.3686
**pM-Stage**	**0.0325**	0.3420	0.7059	1.0000	**0.0073**	0.4886	1.0000	0.7179	0.1394	1.0000
**Budding 10 in 10**	0.1884	0.1299	0.6493	0.5159	**0.0425**	0.2843	**0.0155**	1.0000	0.4960	1.0000
**Chemo**	0.5507	1.0000	0.5225	1.0000	0.6031	0.6031	1.0000	1.0000	0.2692	1.0000
**Radio**	0.7860	1.0000	0.4235	1.0000	0.2098	0.5441	1.0000	0.2689	0.5036	0.3120
**R-Status**	0.6036	1.0000	0.4063	0.2429	**0.0067**	0.1954	0.7955	0.5523	0.9474	0.5031
**ITB**	0.1050	0.0497	0.0092	0.2181	**0.0428**	0.1674	**0.0253**	0.365	0.4174	1.0000
**PTB**	1.0000	0.2615	0.8321	0.6695	**<0.0001**	**0.0035**	**0.0200**	**0.0349**	0.0572	0.4983
**OS**	0.8862	0.7879	0.4252	0.6733	**0.0027**	0.0507	0.0823	0.562	0.9690	0.3469
**DFI**	0.7542	0.8261	0.4481	0.2776	0.2522	0.675	0.3054	0.7789	0.4021	0.6764

L-Stage: lymphatic invasion; V-Stage: venous invasion; R-Status: resection margin status; ITB: intratumoral budding; PTB: peritumoral budding; OS: overall survival; DFI: Disease Free Interval

Supplementary Table 3: Multivariate analysis of CD8 in PDAC

Feature	*P*-value
CD8p/buds	0.5425
pT	0.7289
pN	0.4569
L	0.272
V	0.0096
M	0.0995
R	0.4252
Budding	<0.0001
Chemo	0.0032

L: lymphatic invasion; V: venous invasion; R: resection margin; ITB: intratumoral budding; PTB: peritumoral budding; OS: overall survival; DFI: Disease Free Interval

Supplementary Table 4: Multivariate analysis of immune markers in PDAC

Feature		HR (95%CI)	*P*-value
**FOXP3**	Low	1.0	**0.006**
	High	2.13 (1.2–3.7)	
**CD8**	Low	1.0	0.7977
	High	1.08 (0.62–1.86)	
**iNOS**	Low	1.0	0.8713
	High	1.05 (0.58–1.91)	
**CD163**	Low	1.0	0.2551
	High	0.72 (0.4–1.27)	

Supplementary Table 5: Median normalized values of the immune markers used as cutoff

Marker	Median normalized value
CD8 p	1.1
CD8 i	0.01
CD4 p	5.71
CD4 i	0.1
FOXP3 p	3.15
FOXP3 i	0.15
CD163 p	13.95
CD163 i	0.7
iNOS p	0.03
iNOS i	0.01

p: peritumoral/perilesional; i: intracellular/intratumoral
